# Green preparation and characterization of AGC-ZM-2022 as a novel mesoporous silica material using palmitic acid as a natural template

**DOI:** 10.1039/d2ra06668h

**Published:** 2023-01-18

**Authors:** Zahra Moradi, Arash Ghorbani-Choghamarani

**Affiliations:** a Department of Chemistry, Faculty of Sciences, Ilam University P. O. Box 69315516 Ilam Iran; b Department of Organic Chemistry, Faculty of Chemistry, Bu-Ali Sina University P. O. Box 6517838683 Hamedan Iran a.ghorbani@basu.ac.ir arashghch58@yahoo.com

## Abstract

In this research project, the preparation of a novel mesoporous silica compound (AGC-ZM-2022) using a fatty acid as a template has been reported for the first time. This mesoporous silica compound was designed using palmitic acid as a template, which is one of the most common saturated fatty acids found in animals, plants, and microorganisms. AGC-ZM-2022 mesoporous silica was prepared using tetraethylorthosilicate as a silica source and palmitic acid as a template (instead of traditional templates) through the sol–gel method. The physical properties and structure of AGC-ZM-2022 were studied by FT-IR, SEM, XRD, TEM, and BET techniques.

## Abstract 2

In this research, for the first time, AGC-ZM-2022 (Arash Ghorbani-Choghamarani and Zahra Moradi-2022) has been reported as a novel mesoporous silica compound. In the described approach, palmitic acid was used as a green and natural template.

## Graphical abstract

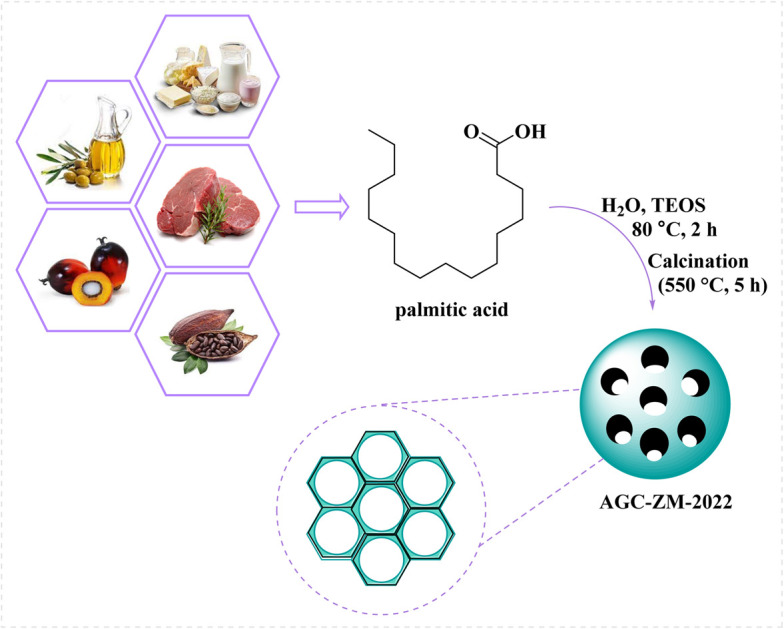

## Introduction

Among the various forms of nanoparticles, nanoporous materials have a special place in chemistry. Nanoporous materials are solid compounds with a pore size of 1 to 100 nm and have a very high available surface area.^1–10^ The structure of these compounds consists of two parts, the wall, and the cavity, in which the sequence of these cavities can continue inside the solid network and they can even communicate with each other within the network. High specific surface area, selectivity, shape, and size are the most important characteristics of these materials, which have led to many applications in different fields such as catalysis, refining, and separation; their role has been highlighted in nanotechnology.^11–15^ The main applications of nanoporous materials in chemistry are their use in the manufacture of chemical sensors, and as a surface for the stabilization of chemical and biochemical catalysts.^16–18^ The development of these materials in the future depends on the manufacture of engineered and controlled porous materials for the intended applications. Zeolites,^19^ MOF,^20^ microspheres,^21^ nanoporous carbon,^14^ and silica^22^ are the most important nanoporous structures.

Fatty acids are hydrocarbon chains of varying lengths and degrees of saturation (dual bonds) with a carboxyl group at one end and a methyl group at the other. Fatty acids are divided into two types, saturated and unsaturated. These natural carboxylic acids could be extracted from a variety of natural sources such as nuts, canola, olive oil, sunflower, sesame, *etc.*^23^

The green chemistry term refers to the design of chemical products and processes that reduce or eliminate the production and use of hazardous substances. Another goal of chemists is the simplicity of synthetic methods and appropriateness in terms of consumption costs. Different and varied methods are presented every day for the synthesis of organic compounds, but finding and using an efficient, useful, scientific and environmentally friendly method is one of the most important features. Therefore, in this research, new compounds of silica nanoparticles based on natural fatty acids have been designed and synthesized, which will open a new way for researchers to use fatty acids to prepare new catalytic supports. Here, a new type of mesoporous silica composite was prepared in a simple way using palmitic acid (a natural fatty acid) as a template. We nominate this new mesoporous material as AGC-ZM-2022 (Arash Ghorbani-Choghamarani and Zahra Moradi – 2022).

## Experimental

### Materials

Palmitic acid, tetraethyl orthosilicate (TEOS), and sodium hydroxide (NaOH) were purchased from Merck company.

### Preparation of AGC-ZM-2022 nanoparticles

**Scheme 1:**
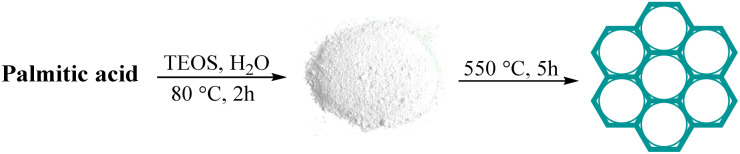
Synthesis of AGC-ZM-2022 nanoparticles.

To prepare AGC-ZM-2022, 2.7 mmol of palmitic acid was added to a solution of deionized water (250 mL) at 80 °C (reflux) under vigorous stirring. Then, tetraethyl orthosilicate (TEOS, 5 mL) was added dropwise and the reaction mixture was stirred for 2 hours. At the end of the mentioned time, the resulting mixture was cooled down to room temperature and the white solid powder was filtered and dried in an oven at 80 °C. The resulting solid powder was calcined at 550 °C for 5 hours at the rate of 2 °C min^−1^ (Scheme 1).

## Results and discussion

FT-IR (BRUKER GERMANY VBRTEX 70), SEM (JOEL JEM- 2010, voltage 200 kW), TEM (TEM Philips EM 208S), XRD (X-ray diffraction (XRD, GBC-Difftech MMA)), and BET (Micromeritice/Asap 2020 V3.03 G) analyzes were applied to identify and study the structure of this new mesoporous silica compound.

**Fig. 1:**
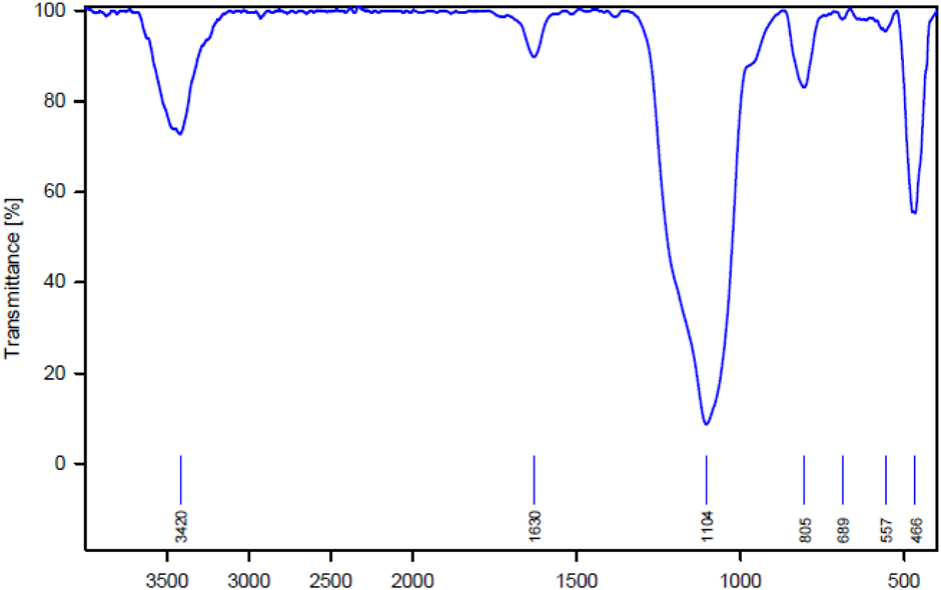
FT-IR pattern of AGC-ZM-2022.

The structure of AGC-ZM-2022 was considered by FT-IR spectroscopy (Fig. 1). The peak in the 466 cm^−1^ regions is related to the Si–O–Si bending vibrations. The peaks in the area of 845 cm^−1^ and 1104 cm^−1^ are also attributed to the symmetric and asymmetric stretching vibrations of Si–O–Si. Also, the stretching vibration in the 3420 cm^−1^ regions is related to the surface hydroxyl.

**Fig. 2:**
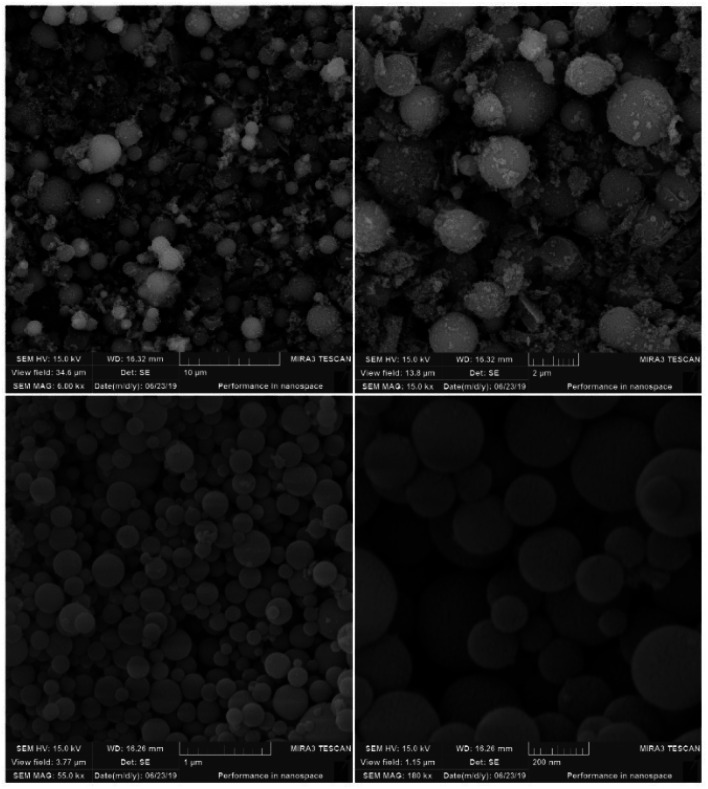
SEM images of AGC-ZM-2022.

The SEM images of the synthesized AGC-ZM-2022 nanoparticles are shown in Fig. 2. The images show that the prepared nanoparticles have a uniform distribution and a spherical structure with particle sizes between 50 to 200 nm.

**Fig. 3:**
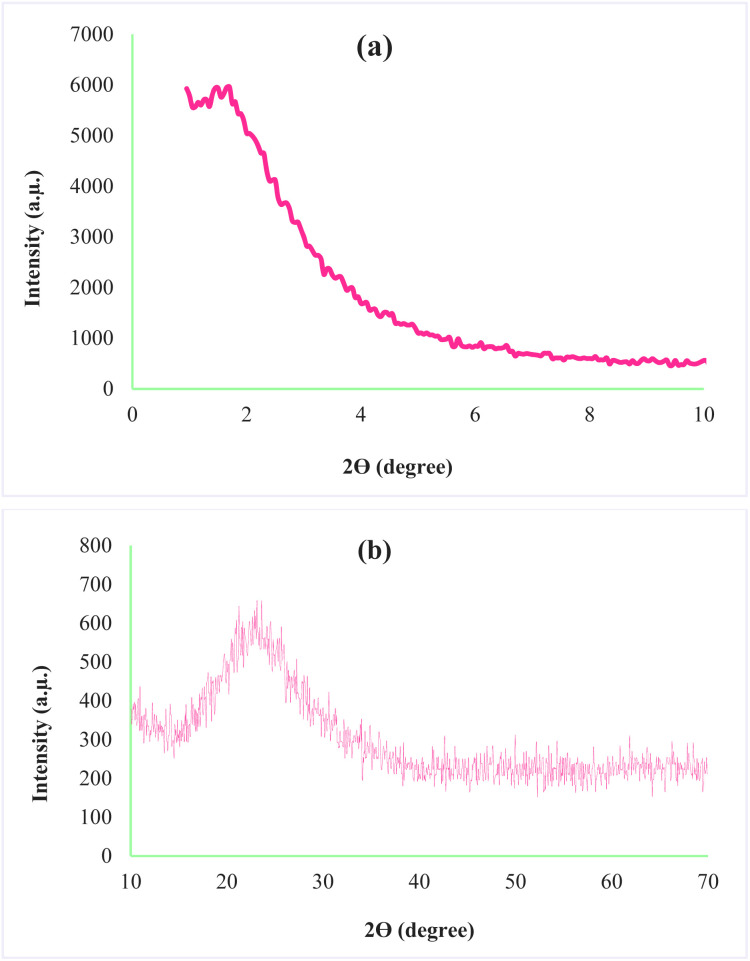
X-ray diffraction of AGC-ZM-2022 (a) low angle, (b) high angle.

X-ray diffraction is one of the analyses used to determine the structural properties of nanoparticles. The XRD pattern for AGC-ZM-2022 nanoparticles is shown in Fig. 3. The low angle diffraction pattern for AGC-ZM-2022 nanoparticles (Fig. 3a) in the range of 0.9–2, is agreeable with the reported XRD of mesoporous compounds in the literature.^24–28^ Due to the high-angle X-ray diffraction (Fig. 3b), there is a wide peak at an angle between 15 and 30, which is attributed to the structure of the mesoporous material.^29,30^

**Fig. 4:**
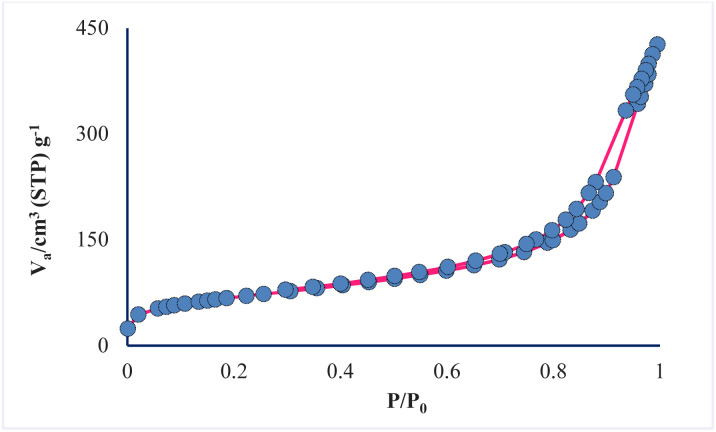
Nitrogen adsorption–desorption isotherm of AGC-ZM-2022 nanoparticles.

Through porosity analysis through N_2_ adsorption–desorption, the IUPAC isotherm type, pore size and surface area of the prepared mesoporous sample can be accessed. The absorption–desorption curve of the mesoporous silica material AGC-ZM-2022 (as shown in Fig. 4), corresponds to the isotherm IV of the IUPAC classification with H3 residue, which is characterized as a uniform mesoporous material. Absorption and resorption branches by a strong bend (*P*/*P*_0_ between 0.3–0.9), showed that the capillary condensation occurred in the uniform mesopore.^31–37^

The prepared mesoporous pores properties were compared with synthesized nano-silica by Amer (2020).^38^ According to the BET data (Table 1), the surface area of the prepared mesopore is 239.1 m^2^ g^−1^, while the surface area of nano-silica is 160.29 m^2^ g^−1^. The results show that the synthesized nanoparticles have good porosity compared to similar reported mesoporous.

**Table 1:** Physicochemical properties of AGC-ZM-2022

Surface area (m^2^ g^−1^)	Mean pore diameter (nm)	Total pore volume (cm^3^ g^−1^)
239.1	10.688	0.6389

**Fig. 5:**
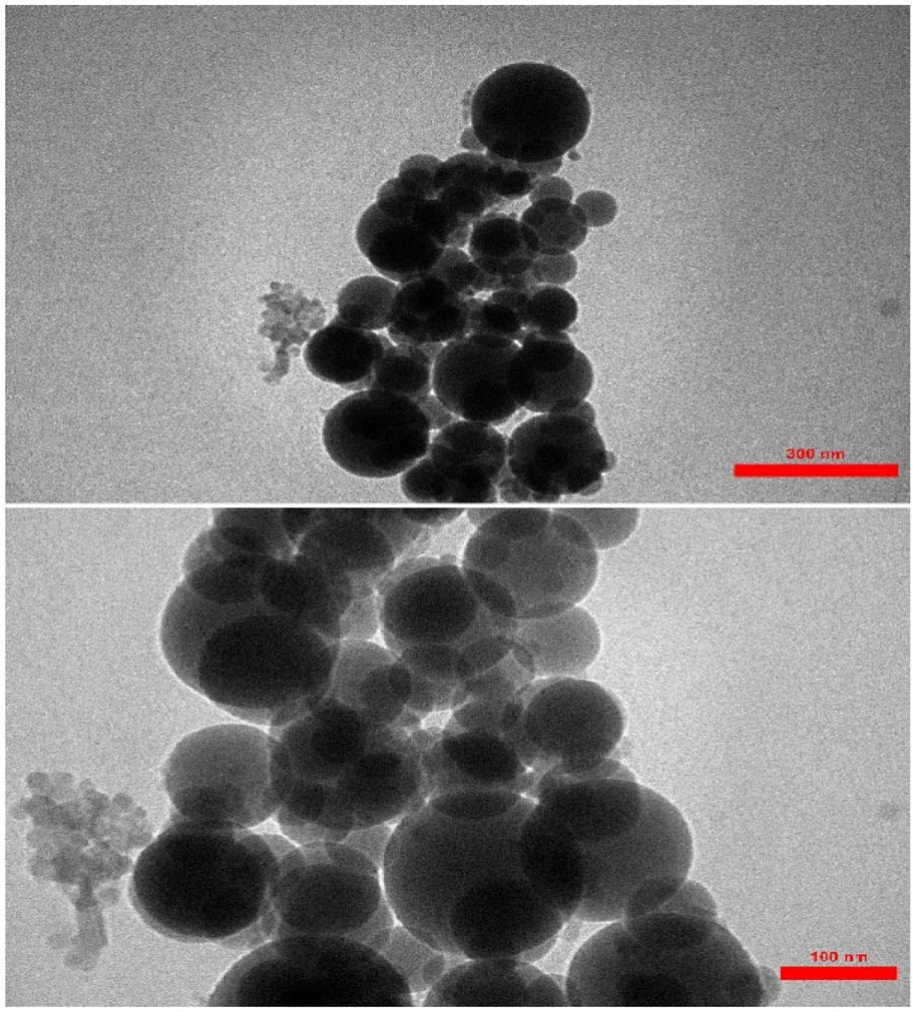
TEM images of AGC-ZM-2022 nanoparticle.

The TEM (transmission electron microscopy) images of the AGC-ZM-2022 nanoparticles are shown in Fig. 5. The images showed a definite spherical shape.

Different conditions to obtain mesoporous silica AGC-ZM-2022 *via* palmitic acid/tetraethylorthosilicate are analyzed in this study (Table 2). To the best of our knowledge, this system has not been reported before. In the first experiment, to a solution of deionized water (500 mL) at a temperature of 80 °C, 3.5 mL of 2 M NaOH solution and 2.7 mmol of palmitic acid were added under vigorous mixing. Once the solution was homogeneous, 5 mL of tetraethyl orthosilicate (TEOS) was added dropwise and the reaction mixture was stirred for 2 h. After the end of the mentioned time, the resulting mixture was cooled down to room temperature and a white powder was obtained, which dried in an oven at a temperature of 80 °C. The obtained solid powder was calcined at 550 °C for 5 hours at the rate of 2 °C min^−1^ but did not yield much efficiency (Table 2, entry 1).

**Table 2:** The reaction conditions for the preparation of AGC-ZM-2022

Entry	Palmitic acid (mmol)	TEOS (mL)	NaOH (2 M) (mL)	*T* (°C)	*V*_H_2_O_ (mL)	Yield (g)
1	2.7	5	3.5	80	500	—
2	2.7	5	—	80	500	Trace
3	2.7	5	—	80	250	1
4	—	5	—	80	250	—
5	2.7	2.5	—	80	250	0.5
6	1.35	5	—	80	250	0.4
7	1.35	2.5	—	80	250	Trace
8	2.7	5	—	90	250	1
9	2.7	5	—	50	250	Trace

In the next experiment, the reaction was performed with the above-mentioned conditions in the absence of sodium hydroxide, which did not yield good returns (Table 2, entry 2). For this reason, to improve the outcome of synthesis, the content of consumed water was reduced to 250 mL, and after calcination, a solid powder was obtained with excellent yield, therefore, the amount of solvent has a significant effect on the synthesis of mesoporous AGC-ZM-2022 (Table 2, entry 3).

However, in the next step to evaluate the effect of palmitic acid in the synthesis of mesoporous AGC-ZM-2022, the reaction was performed without the presence of palmitic acid (250 mL H_2_O 80 °C, 5 mL TEOS) and no yield was obtained, which indicates the presence of palmitic acid is necessary for the preparation of AGC-ZM-2022 (Table 2, entry 4). In another experiment, different amounts of palmitic acid and tetraethylorthosilicate were investigated at different temperatures. The results (Table 2, entries 5–7) show that the low amount of palmitic acid and tetraethylorthosilicate is not effective.

The results show that by increasing the reaction temperature from 80 °C to 90 °C, no significant change in product yield was observed (Table 2, entry 8). By performing the reaction at a temperature of 50 °C, a small yield of the desired mesoporous silica composition was obtained, and the results show that temperature is effective in the synthesis of silica nanoparticles (Table 2, entry 9).

According to the results obtained from Table 2, the amount of 2.7 mmol of palmitic acid, 250 mL of deionized water, and tetraethyl orthosilicate (TEOS, 5 mL) at 80 °C were chosen as the optimal conditions for the preparation of solid powder.

## Conclusions

In conclusion, we have reported the synthesis of a novel mesoporous silica material using a fatty acid as a green natural, and cost-effective template. The desired mesoporous compound was obtained in a simple method using palmitic acid, which can be used as solid support for the heterogenization of homogeneous catalysts. The physical properties and structure of synthesized mesoporous AGC-ZM-2022 were confirmed by several techniques such as FT-IR spectroscopy, X-ray diffraction analysis (XRD), scanning electron microscope (SEM), TEM (transmission electron microscopy), and N_2_ adsorption–desorption.

## Author contributions

Zahra Moradi: methodology, validation, investigation, writing – original draft. Arash Ghorbani-Choghamarani: funding acquisition, supervision, conceptualization, resources, writing – review & editing.

## Conflicts of interest

There is no contrast to declaring conflicts of interest.

## Supplementary Material
